# Secretory Production of Functional Grouper Type I Interferon from *Epinephelus septemfasciatus* in *Escherichia coli* and *Bacillus subtilis*

**DOI:** 10.3390/ijms21041465

**Published:** 2020-02-21

**Authors:** Hsuan-Ju Lin, Joan Tang Xiao Joe, Wen-Jung Lu, Mei-Ying Huang, Ting-Hsuan Sun, Sheng-Pao Lin, Yi-Chuan Li, Ya-Chin Tsui, Ming-Wei Lu, Hong-Ting Victor Lin

**Affiliations:** 1 Department of Food Science, National Taiwan Ocean University, Keelung 20224, Taiwan; angel810801@gmail.com (H.-J.L.); miss350100@gmail.com (W.-J.L.); amysun810922@gmail.com (T.-H.S.); simba32013@yahoo.com.tw (S.-P.L.); dacal852@gmail.com (Y.-C.L.); maggietsui0820@gmail.com (Y.-C.T.); 2 Doctoral Degree Program in Marine Biotechnology, The College of Life Sciences, National Taiwan Ocean University, Keelung 20224, Taiwan; Joan94_0137@live.cn; 3 Doctoral Degree Program in Marine Biotechnology, Academia Sinica, Taipei 11529, Taiwan; 4 Division of Aquaculture, Fisheries Research Institute, Council of Agriculture, No. 199, Hou-Ih Road, Keelung 20246, Taiwan; myhuang@mail.tfrin.gov.tw; 5 Department of Aquaculture, National Taiwan Ocean University, Keelung 20224, Taiwan; 6 Center of Excellence for the Oceans, National Taiwan Ocean University, Keelung 20224, Taiwan

**Keywords:** *Epinephelus septemfasciatus*, interferon, nervous necrosis virus, signal peptide, aquaculture

## Abstract

Nervous necrosis virus (NNV) results in high mortality rates of infected marine fish worldwide. Interferons (IFNs) are cytokines in vertebrates that suppress viral replication and regulate immune responses. Heterologous overexpression of fish IFN in bacteria could be problematic because of protein solubility and loss of function due to protein misfolding. In this study, a protein model of the IFN-α of *Epinephelus septemfasciatus* was built based on comparative modeling. In addition, PelB and SacB signal peptides were fused to the N-terminus of *E. septemfasciatus* IFN-α for overexpression of soluble, secreted IFN in *Escherichia coli* (E-IFN) and *Bacillus subtilis* (B-IFN). Cytotoxicity tests indicated that neither recombinant grouper IFN-α were cytotoxic to a grouper head kidney cell line (GK). The GK cells stimulated with E-IFN and B-IFN exhibited elevated expression of antiviral Mx genes when compared with the control group. The NNV challenge experiments demonstrated that GK cells pretreated or co-treated with E-IFN and B-IFN individually had three times the cell survival rates of untreated cells, indicating the cytoprotective ability of our recombinant IFNs. These data provide a protocol for the production of soluble, secreted, and functional grouper IFN of high purity, which may be applied to aquaculture fisheries for antiviral infection.

## 1. Introduction

*Epinephelus*, one of the largest genera of groupers in the family *Serranidae*, is a teleost fish often found around reefs. *Epinephelus* can be quite large (over a meter and a hundred kilograms), which makes them a commercially viable and valuable food source. However, a number of grouper fish species are endangered due to overfishing and poor management of coral reef fisheries. The degradation of the grouper’s natural environment necessitates the rapid development of a grouper aquaculture industry. One of the major challenges in grouper aquaculture is viral infections caused by the red-spotted grouper nervous necrosis virus (RGNNV) [1,2,3,4], which is a non-enveloped icosahedral RNA piscine nodavirus containing two-single-stranded RNA [5,6], resulting in high mortality rates (80–100%) of affected larvae, juveniles, and some adults in >120 species of cultured marine fish worldwide [7,8,9].

Huge losses occur worldwide in the grouper aquaculture industry every year due to NNV infection [2,9]. Although various vaccines have been developed, such as formalin-in activated and virus-like particle vaccines [10,11,12,13], it might be relatively difficult to inject the vaccines into grouper larvae and juveniles [14], which are the primary targets of RGNNV infections. Another approach to reduce RGNNV is interferon (IFN) introduction to fish, which was reported to prevent viral infection in vivo and in vitro [3,15,16,17,18]. IFNs are secretory cytokines in vertebrates involved in the inhibition of virus replication and modulation of immune responses [19]. To date, three types of IFNs have been identified (type I, II, III): Type I/III IFNs are induced by viral infections, whereas type II IFNs are induced by mitogenic or antigenic stimuli [19,20]. Upon viral infection, infected cells secrete type I IFN α/β, and these IFNs are believed to protect other unaffected cells from viral infection by inducing the expression of many genes, some of which encode antiviral proteins, such as double-stranded RNA (dsRNA)-activated protein kinase [21], 2′,5′-oligoadenylate synthetase (OAS) [22], and Mx proteins [23].

For commercial and industrialized purposes, recombinant IFNs from *Epinephelus septemfasciatus* have been produced and were shown to up-regulate *mx* expression and provide protection against NNV [15]. The grouper and salmon IFNs could up-regulate Mx gene expression in grouper kidney (GK) cells and show antiviral activities in Malabar grouper larvae infected with NNV [3], and pretreatment of IFN-α2a at 48 h prior to NNV infection could significantly increase the survival rate of zebrafish [24]. However, overexpression of eukaryotic IFNs in *E. coli* could be challenging as IFNs may not fold properly and precipitate into inclusion bodies during expression [3,25,26]. Solubilization of IFNs from inclusion bodies using denaturing agents or detergents have been adapted to refold and recover the biological activities of recombinant IFNs, but it is time-consuming, not environmentally-friendly, and is relatively ineffective. In addition, the recovery of cytosolic recombinant IFNs requires additional processing, such as cell disruption and protein purification [3], which increases production costs and time. In this study, a cell-disruption-free strategy for the production of soluble recombinant grouper IFN has been investigated and biological activities of the IFN were evaluated.

## 2. Results and Discussion

### 2.1. Multiple Sequence Alignment and Protein Modeling of Grouper IFN-α

**Figure 1:**
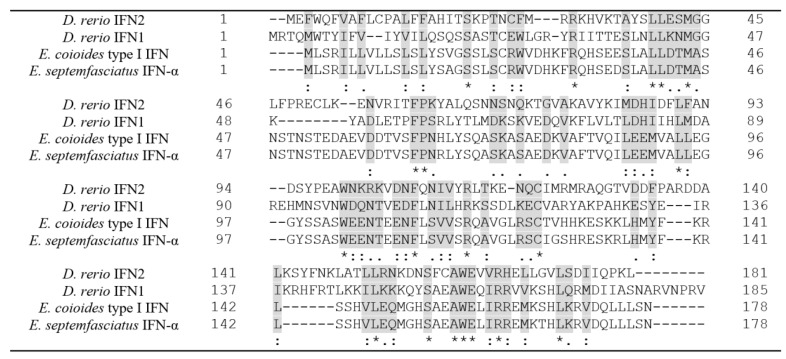
Multiple alignment of the interferons from *Danio* and *Epinephelus*. Multiple alignment of amino acid sequences of Interferon (IFN)2 from *D. rerio* (UniProtKB: A8E6E2), IFN1 from *D. rerio* (UniProtKB: Q8AY12), type I IFN from *E. coioides* (UniProtKB: M9WVF6), and IFN-α from *E. septemfasciatus* (UniProtKB: E9RH07). The different sequences have been submitted to a multiple alignment using the Clustal. The following three characters are used to highlight conserved amino acids: ‘*’ indicated positions which have a single, fully conserved residue; ‘:’ indicated conservation between groups of strongly similar properties; ‘.’ indicated conservation between groups of weakly similar properties.

IFNs are small helical cytokines in vertebrates that are involved in inhibition of virus replication and modulation of immune responses [18]. Using Clustal [27], multiple sequence alignment of the *D.* *rerio* IFN1 and IFN2, *E. coioides* IFN, and *E. septemfasciatus* IFN was performed, as shown in Figure 1.

Nineteen of the 178 amino acid residues in *E. septemfasciatus* IFN were indicated as the fully conserved residues in the aligned protein sequences (the ‘*’ symbol). BLASTP [28] indicated that the IFN-α from *E. septemfasciatus* (178 amino acid residues) shares a 33% identity with IFN1 (185 amino acid residues) from *Danio rerio* (zebra fish), whose protein structure contains one disulfide bridge [29]; In addition, the IFN1 from *D. rerio* is the protein with a determined 3D structure which shares the best sequence identity with the IFN-α from *E. septemfasciatus*. BLASTP [28] indicated that IFN-α from *E. septemfasciatus* shares 23% identity with IFN2 (181 amino acid residues) from *Danio rerio*. BLASTP [28] determined that the IFN-α from *E. septemfasciatus* shares 94% identity with type I IFN (178 amino acid residues) from *Epinephelus coioides*. In IFN2 from *Danio rerio,* Glu163(F) interacts with both helices C and E, while Val165(F) and Arg166(F) interact with both the D helix and the AB loop [29]. It is possible that the corresponding amino acid residues in *E. septemfasciatus* IFN-α have the same roles in protein folding.

**Figure 2:**
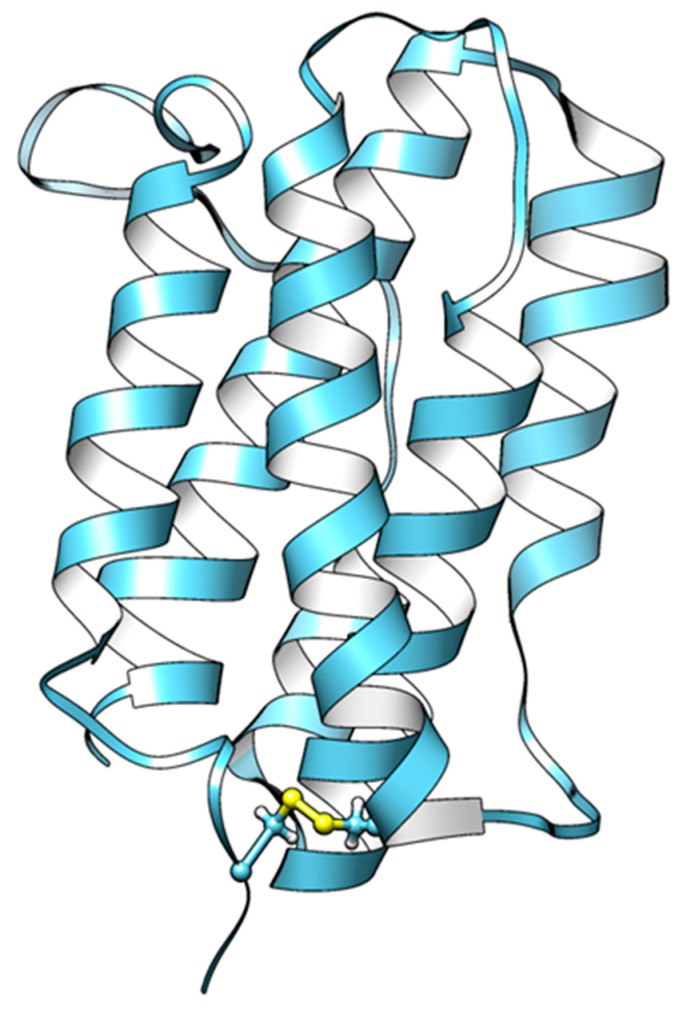
Protein model of the type I IFN-α from *E. septemfasciatus*. The IFN-α model was built based on the 3D structure of the interferon 1 from *Danio rerio* (PDB 3PIV) by using Chimera and Modeller.

A protein model of the IFN-α of *E. septemfasciatus* was built based on comparative modeling by using Chimera [30] and Modeller [31], as shown in Figure 2. The IFN-α of *E. septemfasciatus* shares a sequence identity of 33% with the interferon 1 of zebrafish (*Danio rerio*), with an E value of 2 × 10^−29^. The protein structure modeling of the mature form (removal of the predicted signal peptide) of IFN-α of *E. septemfasciatus* (Δ20IFN-α) was built based on the 3D structure of the zebrafish interferon 1 (PDB databank 3PIV) [29], whose 3D structure was solved using X-ray diffraction with a resolution of 2.09 Å. The model we built for *E. septemfasciatus* IFN-α had a GA341 of 1.00, zDOPE of −1.56, estimated RMSD of 1.544, and estimated overlap (3.5 Å) of 0.943. Results for models with a GA341 greater than 0.7 and negative zDOPE value are relatively reliable [30,32].

As shown in Figure 2, six α-helices arranged in an antiparallel bundle manner to form the core structure were observed in the IFN-α model. As expected, this model contains one intramolecular disulfide bridge (as shown in yellow) to connect the N-terminal helix A to helix D, and the helix F may interact directly with the receptor [29]. A larger loop was observed between helix A and helix B in the protein modeling *E. septemfasciatus* IFN-α (amino acid residues 44 to 64) as compared to the protein structure of interferon 1 from zebrafish.

### 2.2. Strategy for Production of Secreted IFN-α in E. Coli and B. Subtilis

The type I IFN-α from *E. septemfasciatus* has two cysteines at position 23 and 125 of the polypeptide chain. Disulfide bond is crucial for the stability of final protein structures by decreasing the conformation freedom of the random coils, and the entropy for the reaction to ordered, native 3D structure of a protein is less negative in the presence of a disulfide bond. As a result, mispaired cysteine residues could therefore lead to misfolding and non-functional proteins [33]. Previous studies of IFN indicate that this disulfide bond may alter activity [34,35,36], and mispaired cysteines can cause misfolding, aggregation and ultimately result in low active protein yields [37,38,39]. This study aimed to overexpress a functional, soluble IFN-α from *E. septemfasciatus* in bacteria hosts, such as *E. coli* and *B. subtilis*. Our strategy was to translocate the recombinant IFN-α from the cytosol, a reducing environment, into the cell periplasm (*E. coli*) or extracelluar space (*B. subtilis*), both of which are oxidizing compartments; suitable for the disulfide-bond formation, which could benefit the folding, stability and solubility of IFN-α. Previous reports demonstrate that disulfide oxidoreductases and isomerases located in the *E. coli* periplasm catalyze the formation of disulfide bonds enabling the accumulation of properly folded, soluble protein; making the periplasm an ideal compartment for the expression of therapeutic proteins [38]. In addition, a gene cluster which putatively encodes disulfide oxidoreductases for the catalysis of disulfide bonds has been reported in *B. subtilis,* although their substrates are unknown [40].

**Figure 3:**
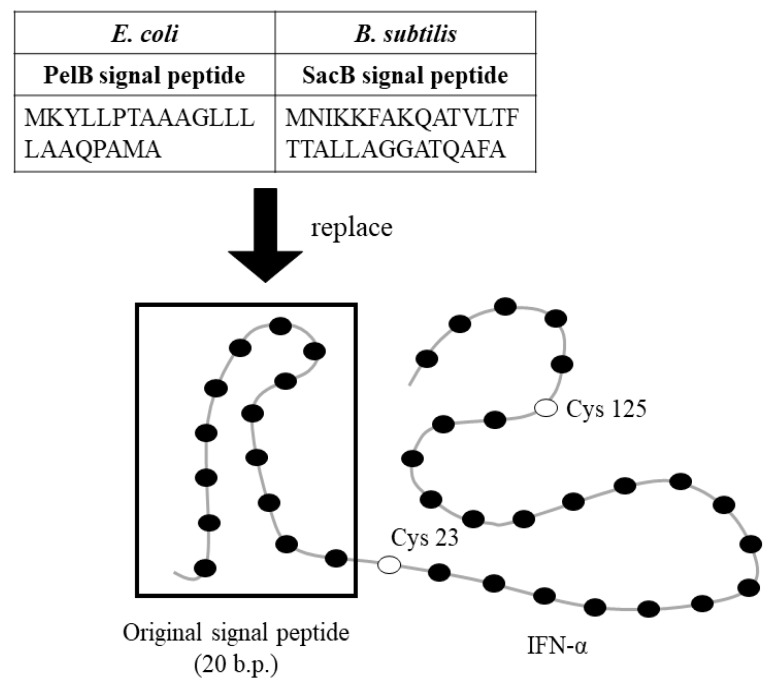
The design of recombinant IFN construction.

IFN-α from *E. septemfasciatus* is predicted to have signal peptides in the N-terminus [15,41]. In this study, we used the SignalP 4.1 Server to predict the location and length of the signal peptide in the *E. septemfasciatus* IFN-α [42]. The results indicate that a signal peptide is contained in the first 20 amino acids of the N-terminus of IFN-α, similar to type I interferon from *E. coioides* [41]. The mature form of IFN-α (Δ20) overexpression in *E. coli* was fused, in the N-terminus, with the PelB signal peptide from the pectatelyase B of *Erwinia carotovora* (Peptide sequence: MKYLLPTAAAGLLLLAAQPAMA) (Figure 3), which translocates recombinant IFN-α into periplasm space of *E. coli* during protein overexpression. In addition, the mature form of IFN-α (Δ20) overexpression in *B. subtilis* was fused, in the N-terminus, with a SacB signal peptide from the levansucrase of *B. subtilis* (Peptide sequence: MNIKKFAKQATVLTFTTALLAGGATQAFA) (Figure 3), which would lead recombinant IFN-α excretion into the extracellular space during protein production.

### 2.3. Effect of Inducing Time for IFN-α Overexpression

Pilot experiments were adapted to evaluate IFN-α overexpression at 18, 24, 30, 37, and 42 °C. Cells at 24 °C have the highest overexpression of IFN-α (data not shown); therefore, 24 °C was used for all further experiments. The recombinant IFN-α overexpression was further investigated at different induction time (3, 6, 9, and 12 h). *E. coli* cells harboring pET-IFN plasmids were induced for various times at 24 °C. Cells were harvested at each time point for protein purification. Soluble IFN-α was obtained from the cells using osmotic shock and purified using affinity chromatography. 

**Figure 4:**
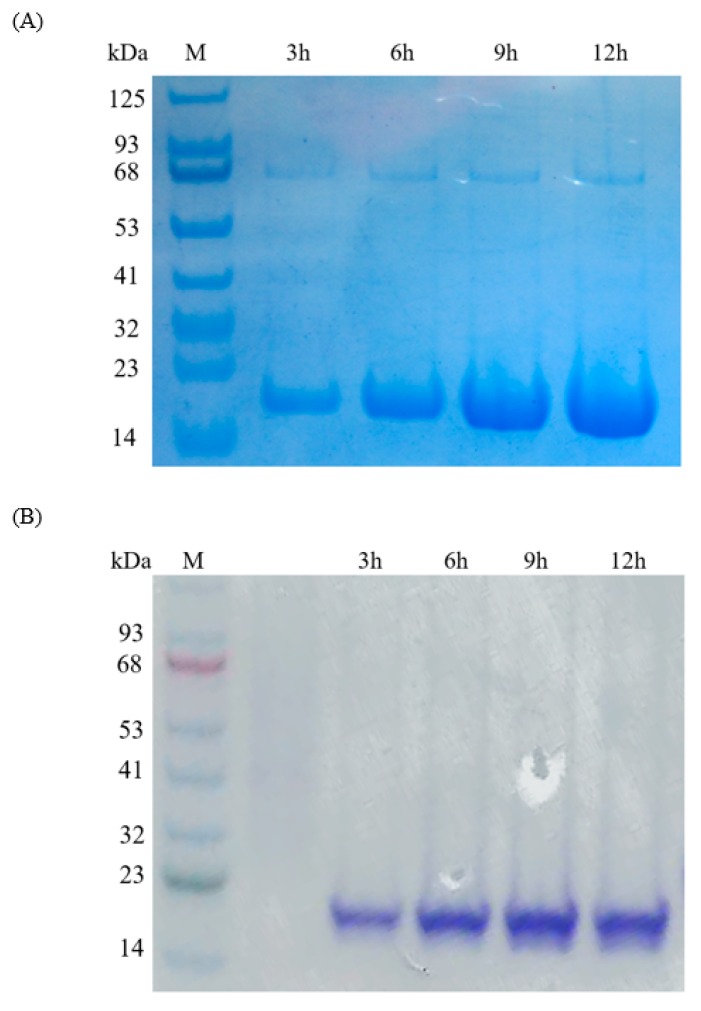
SDS−PAGE of the purified IFN-α from (**A**) *E. coli* and (**B**) *B. subtilis* overexpression systems at various induction time. Lane M, protein marker. *E. coli* and *B. subtilis* cells harboring plasmids encoding IFN were induced by 0.2 mM IPTG at 24 °C for 3, 6, 9, 12 h.

The overexpression of IFN-α, with an estimated molecular weight of 18 kDa, was analyzed by using gel-electrophoresis and the highest level of IFN-α expression in *E. coli* cells was observed at 12 h (Figure 4A); *E. coli* cells tended to lyse when the induction time exceeded 15 h. *B. subtilis* harboring pHT-IFN plasmid was induced at 3, 6, 9, and 12 h at 24 °C; cells were harvested at each time point for protein purification. Soluble IFN-α was collected from the cell culture supernatant and purified using affinity chromatography. IFN-α was analyzed using gel-electrophoresis, with the IFN-α peaking at 9 h (Figure 4B). Both proteins were confirmed by either Western blot or peptide mass fingerprinting (data not shown). Periplasmic and extracellular secretion of overexpressed proteins offer several key advantages for protein production, for example, a better target protein purity due to the reduction impurities from other bacterial proteins and nucleic acids [43], and an oxidizing environment, such as periplasm and extracellular space, which facilitates disulfide bond formation for correct protein folding [44]. As shown in Table 1, overexpressed fish interferons in bacterial hosts can form inclusion bodies or insoluble forms, thus requiring further recovery procedures, such as protein denaturation and refolding, to obtain functional interferons. Kakeshita, et al. [45] exploited the AmyE signal peptide in a *B. subtilis* system for human hIFN-α2b overexpression with a yield of 1.64 mg/L. In addition, modifications of in the C-terminus of SecA protein in *B. subtilis* overexpression hosts enhanced the extracellular levels of hIFN-α2b up to 2.2-fold. In this study, overexpression, extraction, and purification of IFN-α from the periplasmic space of *E. coli* gave a yield of 6 mg per L culture. Recovery and purification of IFN-α from the growth media of *B. subtilis* produced 1.3 mg per L culture. Other strategies, such as co-expression of chaperons and the construction of IFN fused with soluble tag proteins (to increase the solubility of recombinant IFN) have been tested [46,47]. Co-expression of *E. coli* chaperones, such as GroEL/ES, failed to increase IFN solubility at 37 °C but managed to decrease the inclusion body formation up to 62% at 25 °C [46]. Fusing IFN with a soluble tag (e.g., Small Ubiquitin-like Modifier; SUMO) could improve the solubility of IFN, but the removal of a large, soluble tags requires additional protein purification steps [47].

### 2.4. Cytotoxicity of the Recombinant IFN-α Against Grouper Head Kidney Cell

**Figure 5:**
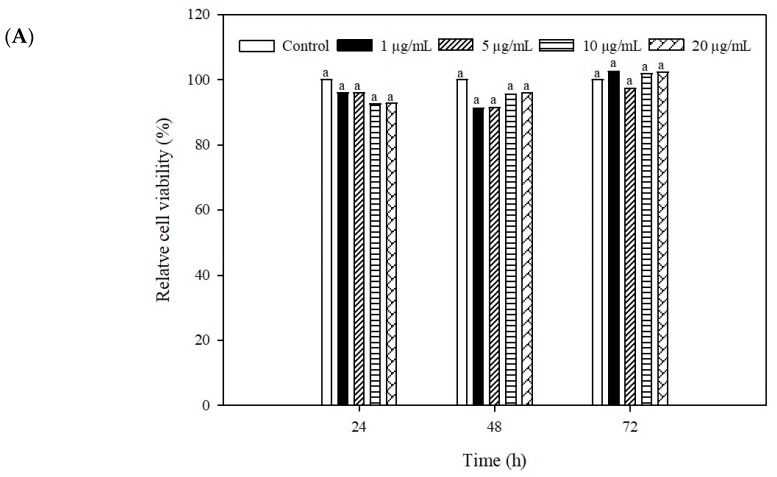

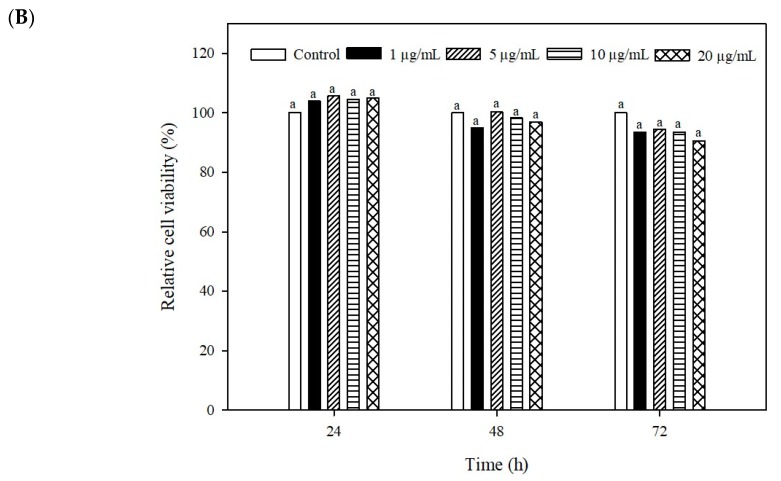
Cytotoxicity of the IFN-α overexpressed in (**A**) *E. coli* (**B**) *B. subtilis* against grouper kidney (GK) cells by MTT assay. The GK cells were incubated with E-IFN for 24, 48, and 72 h prior to MTT assay. The O.D. at 540 nm of each group was compared to the untreated control cells. Data are expressed as mean ± SD (n = 3). Different letters at the top of the bars are significantly different (*p* < 0.05).

Cytotoxicity of recombinant IFN-α was evaluated by using MTT assays. Grouper head kidney GK cells incubated with E-IFN at all tested concentrations (1, 5, 10, 20 μg/mL) exhibited more than 85% cell viability at 24 and 48 h and more than 95% at 72 h (Figure 5A). GK cells incubated with B-IFN at all tested concentrations had more than 90% cell viability 24, 48, and 72 h (Figure 5B). According to ISO 10993-5, neither IFN would be considered to be cytotoxic as cell viability is greater than 70% [48]. In addition, phase-contrast microscopic images of GK cells incubated with recombinant IFN-α from both *E. coli* and *B. subtilis* had no significant differences in cell morphology or growth when compared to control group (data not shown).

Kuo [3] compared the anti-NNV effects of different recombinant fish type I interferons (*E. septemfasciatus*, gIFN; *Salmo salar*, Sifn; *Dicentrarchus labra*, sbIFN; *Oreochromis niloticus*, tpIFN). In cytotoxicity tests, the cell survival rate of gIFN, sIFN, sbIFN, and tpIFN at 10 μg/mL was reduced by approximately 4.3-, 6.0-, 2.0-, and 2.5-fold when compared with control. Our data indicates that secreted E-IFN and B-IFN at up to 20 μg/mL had very little cytotoxic effects in GK cells, indicating that this strategy could be effective for IFN production lacking cytotoxic properties.

### 2.5. Recombinant IFN-α Stimulated Mx Gene Expression

RT-PCR experiments were used to confirm whether E-IFN and B-IFN could induce expression of the Mx protein, a member of the GTPase family with antiviral functions [49]. GK cells were treated with 1 μg/mL of E-IFN and B-IFN, individually, for 3, 6, and 12 h, followed by total RNA extraction and analysis of *Mx* gene expression using RT-qPCR.

**Figure 6:**
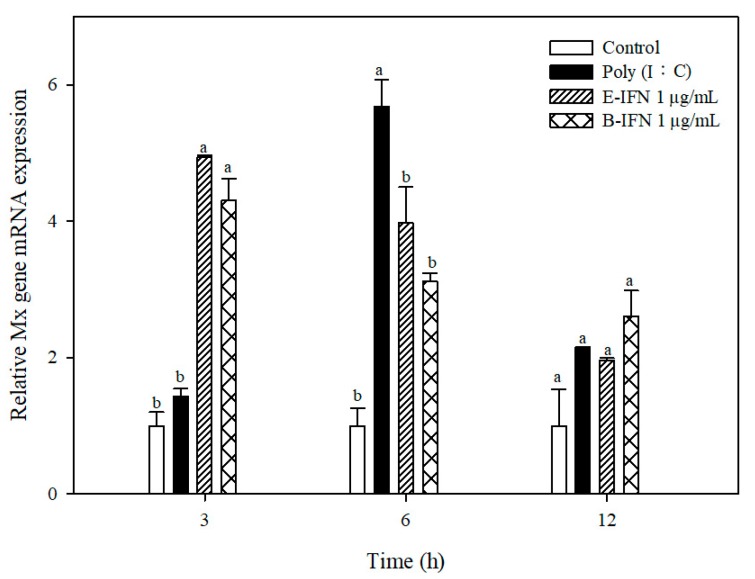
*Mx* gene expression in the GK cells treated with recombinant IFNs. *Mx* gene expression at 3, 6, and 12 h in GK cells treated with 50 μg/mL of poly(I:C) or 1 μg/mL of E-IFN and B-IFN were measured by real-time PCR. The results were normalized against β-actin gene and data of triplicate samples were expressed as the mean fold increase relative to the control. Cells cultured with medium; 2^–ΔΔCT^ method. Data are expressed as mean ± SD from triplicate experiments. Different letters in each grouped bars indicated significant differences (*p* < 0.05).

We observe that E-IFN increases *Mx* gene expression by 5-, 4-, and 2-fold at 3, 6, and 12 h, respectively; in addition, B-IFN increases *Mx* gene expression by 4.2-, 3.2-, and 2.5- fold at 3, 6, and 12 h, respectively (Figure 6). The peak expression of *Mx* gene was achieved when GK cells were incubated with B-IFN and E-IFN for 3 h, followed by decreased expression at 6 and 12 h. These results demonstrate that E-IFN and B-IFN can stimulate GK cells to express the *Mx* gene.

Previous results demonstrate that functional recombinant IFNs increase *Mx* gene expression. GK cells treated with recombinant Sevenband grouper IFN (SgIFN) for 3, 6, and 24 h increased *Mx* gene expression by 6-, 3.7-, and 2.1-fold, respectively [15]. Mandarin fish cells (MFF-1 cells) incubated with recombinant zebrafish type 1 interferon for 4, 12, and 24 h also increase Mx gene expression by 4-, 16-, and 7-fold at 4, 12, and 24 h.

### 2.6. Antiviral Effects of Recombinant IFN in GK Cells

To determine whether E-IFN and B-IFN inhibit viral infection, antiviral tests against NNV were performed. Thus, antiviral experiments using GK cells were performed using recombinant IFN at different stages, pre-treatment and co-treatment, with NNV infection. GK cells in the pretreatment group were incubated with E-IFN or B-IFN for 24 h prior to NNV infection. GK cells in the co-treatment group were incubated with IFN and NNV simultaneously. The relative survival rates of the GK cells were evaluated by using an MTT assay at 24 and 48 h after exposure to virus.

**Figure 7:**
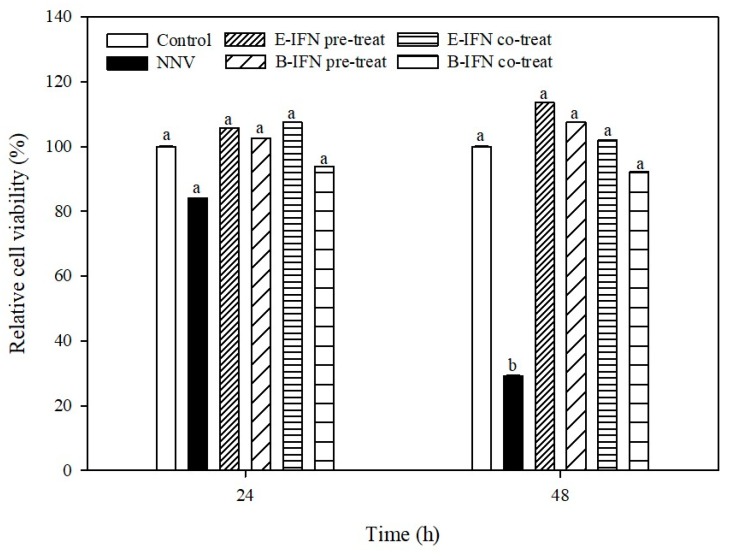
The antiviral effects of the IFNs overexpressed in *E. coli* and *B. subtilis* in the pre-treatment and co-treatment to the GK cells. The GK cells were pre-treated and co-treated with NNV (10^4.5^ TCID50/0.1 mL, 1:100 diluted, 100 μL/per well) and IFN at 1 μg/mL for 24 and 48 h. The cell viability was determined by MTT assay. Data are expressed as mean ± SD of triplicate samples, and Different letters in each grouped bars indicated significant differences (*p* < 0.05).

As shown in Figure 7, GK cells pretreated with 1 μg/mL E-IFN and B-IFN exhibited significantly higher relative survival rates at 24 and 48 h after exposure to NNV when compared with untreated cells. GK cells pretreated with E-IFN or B-IFN showed no significant differences on cell survival rates when compared with control cells (No NNV infection) at 48 h after exposure to NNV, while untreated cells exhibited a relative survival rate of 29% when challenged with NNV. GK cells treated with E-IFN or B-IFN with NNV had significant differences in survival rates when compared with untreated cells. GK cells co-treated with E-IFN and B-IFN exhibited relative survival rates of more than 80% at 24 and 48 h when compared with the control group.

Kuo’s studies demonstrated that gIFN, sIFN, sbIFN, tpIFN (*Epinephelus septemfasciatus*, gIFN; *Salmo salar*, sIFN; *Dicentrarchus labra*, sbIFN; *Oreochromis niloticus*, tpIFN) could protect GK cells from NNV infection. In their pretreatment group, cell survival rate at 24 h was more than 50%; sIFN, gIFN, and tpIFN had poor cell survival rates (~25%) at 48 h. In the co-treat group, the cell survival rate of all groups were more than 80% after 24 h. At 48 h, sIFN, gIFN, and tpIFN cell survival rate was reduced to ~50%. The results indicated that type I interferon could protect GK cells from virus infection [3]. Ooi, et al. [50] used *E. coli* to express recombinant *Atlantic salmon* type 1 interferon (rSasaIFN-α2). In cytopathic experiments, 0.78 ng/mL rSasaIFN-α2 protected cells from virus attack with a cell survival rate greater than 50%. Our data demonstrates that GK cells pretreated or co-treated with our recombinant IFN-α possess higher antiviral activities, indicating that this protocol could be effective when applied to aquacultures for antiviral infections.

## 3. Materials and Methods

### 3.1. Virus, Bacterial Strains, and Media

*E. coli* C43 (F^–^ *ompT gal dcm hsdSB*(r_B_^–^ m_B_^–^)) [51] and *B. subtilis* WB800N (Mobitec GmbH, Göttingen, Germany) was used for protein expression. Luria-Bertani (LB) broth (Difco) was used in bacterial culture. The grouper head kidney cell line (GK cell line) established from the kidney tissue of the yellow grouper *Epinephelus awoara* was maintained at 28 °C in Leibovitz’s L-15 medium (L-15; Gibco, Waltham, MA, USA) supplemented with 10% fetal bovine serum (FBS) (Thermo Fisher Scientific, Waltham, MA, USA) [52]. NNV was originally isolated from a diseased Malabar grouper (*Epinephelus malabaricus*) in a hatchery in southern Taiwan and was propagated on the grouper fin (GF-1) cell line in L-15 media with 5% FBS at 28 °C [53].

### 3.2. Cloning of ifn

The cDNA of *ifn* gene was cloned from *E. septemfasciatus* mRNA by using reverse transcription PCR. The *ifn* gene amplified by using the primers 5′-AAAACCGGATCCGAGCAGTCTGAGCTGTCGTTGG-3′ and 5′- AAACCCCTCGAGGTTGCTCAGCAGCAGCTGGT-3′ for *E. coli* and 5′- AAAACCGGATCCATGAGCAGTCTGAGCTGTCGTTG-3′ and 5′- AAAACC TCTAGAGTTGCTCAGCAGCAGCTGGT-3′ for *B. subtilis*. The *ifn* gene was digested with BamHI and XhoI restriction enzymes and inserted into pET26b vector (Novagen) for *E.coli* and pHT254 vector (Mo Bi Tec) for *B. subtilis* at the BamHI-XhoI site. pHT254-IFN recombinant vector was inserted with the nucleotide sequence (ATG AAC ATC AAA TTC GCA AAA CAA GCA ACA GTT CTT ACA TTC ACA GCA CTT GCA GGC GCA ACA CAA GCA TTC GCA) [54] which encode the signal peptide from the levansucrase of *B. subtilis* before the IFN-α gene for extracellular production of recombinant IFN-α. The recombinant vector encoding IFN was transformed into *E. coli* C43(DE3) and *B. subtilis* WB800N (Mo Bi Tec) for protein overexpression.

### 3.3. IFN-α Overexpression and Purification from E. coli and B. Subtilis

The optimum conditions for the overexpression of grouper rIFN was investigated at various overexpression time (3, 6, 9, and 12 h). The *E. coli* C43(DE3) and *B. subtilis* WB800N harboring plasmid encoding IFN was overexpressed upon IPTG induction at optimum conditions, and followed by osmotic shock. The cells were collected by using centrifugation and re-suspended in the Tris-HCl buffer containing 20% sucrose and EDTA, pH 8.0 (Osmotic shock buffer 1), and the cells were pelleted again and re-suspended and incubated in the Tris-HCl buffer containing 5 mM MgSO4 (Osmotic shock buffer 2) for 30 min. The recombinant IFN was released into the buffer and purified by using a Nickel affinity column (Hitrap chelating column, GE Healthcare), where necessary.

### 3.4. Protein Quantification, Western Blot, and Peptide Mass Fingerprinting

Protein quantification was accomplished using Bradford method [55]. Ten microliters of protein sample was mixed with 200 μL of Bradford dye reagent concentrate (Bio-Rad) on a 96-well plate. The plate was kept in dark for 30 min for color development, followed by the absorbance measurement at 595 nm. A calibration curve was generated using bovine serum albumin as a standard, and made by plotting the concentration against the OD595 and fitting the data by linear regression. Expression of the IFN was analyzed using SDS-PAGE. After separation proteins from the gels were transferred to a PVDF (Polyvinylidene difluoride) membrane and probed with a mouse anti-polyhistidine primary antibody. The PVDF membrane was then incubated with a goat anti-mouse IgG-AP (alkaline phosphatase) conjugate as a secondary antibody, and the proteins were identified by using a colorimetric AP conjugate substrate kit (GE Healthcare). Protein LC-MS analyses were performed on a Waters Acquity nano-UPLC in line with a Waters G2 Q-TOF mass spectrometer. Trypsin-digested protein samples (10 μg/mL) were directly infusion onto mass spectrometer through a syringe pump with flow rate 1 μL/min. The G2 Q-TOF mass spectrometer was run in positive ion, high resolution mode with detection in the range of 600 to 2300 m/z. Source parameters were as follows: capillary voltage, 2.50 kV; source temperature, 90 °C; desolvation temperature, 200 °C; cone gas flow: 20 L/h; the desolvation gas flow, 500 L/h. The protein peak was deconvoluted by the MassLynx MaxEnt1 function according to the following parameters: output resolution, 1.0 Da/channel; output mass range, 35–85 kDa; uniform Gaussian width at half height, 0.75 Da; minimum intensity ratios, 30% for left and right; iteration to convergence for completion.

### 3.5. Mx Gene Expression in the Cells

The head kidney cells (GK cells) from *E. awoara* were incubated with 1 μg/mL purified recombinant IFN expressed from *E.coli* and *B. subtilis*, and polyinosinic:polycytidylic acid (poly(I:C)) (50 mg/mL) (Sigma-Aldrich, St. Louis, MO, USA), a positive control, individually. Total RNA was extracted using the TRIzol reagent (Invitrogen, Carlsbad, CA, USA), and the first strand cDNA was synthesized from 1 mg of total RNA using the Hi-Script I reverse transcriptase (Bionovas, Toronto, ON, Canada). Real-time PCR was conducted to quantify *Mx* gene expression in the IFN-treated cells compared to the control. The assay was performed on a 7500 Real-Time PCR System (Applied Biosystems, Foster City, CA, USA) using Mx and b-actin gene-specific primers. The real-time PCR reaction was performed with an initial denaturing step of 95 °C for 10 min, followed by 40 cycles of 95 °C for 15 s and 60 °C for 1 min, and a 1 cycle melting step. Relative expression of the *Mx* gene was normalized to orange-spotted grouper b-actin and calculated as 2^−∆∆CT^. All of the sample was repeated in triplicate for each time point.

### 3.6. Cytotoxicity Test and Antiviral Effect of IFN

The cultured GK cells were washed with trypsin, and the cell suspension was mixed with L-15 (2% FBS) and cultured in a 96-well plate for one day with a cell density of 300,000 cells/well. After incubation, 10 μL of different concentrations (1, 5, 10, 20 μg/mL) of IFN was added to each well. The cell viability at 24, 48, 72 h was analyzed by MTT assay. To examine the antiviral effect of IFN, GK cells were pre-treated with 1 μg/mL IFN for 24 h prior to NNV challenge [at a multiplicity of infection (MOI) of 1]. For the co-treatment group, the GK cells were co-treated with 1 μg/mL IFN and NNV simultaneously in 12-well plates. Cells incubated with culture medium without any treatment and infected with NNV were used as the control and positive control, respectively. The reduction of cytopathic effects (CPE) following IFN pre- and co-treatment (1 μg/mL) in GK cells infected with NNV at MOI of 1 in 96-well plates were examined using the MTT assay. During the 48 h incubation period, the cell morphology was examined daily using a phase contrast microscope and images were obtained to document the appearance of cytopathic effects. All of the sample was repeated in triplicate at each time point.

### 3.7. Statistical Analysis

Data were statistically analyzed using SPSS Version 12.0 (SPSS Inc., Chicago, IL, USA). One-way analysis of variance (ANOVA) was used to determine the statistical differences between the sample means, with the level of significance set at *p <* 0.05 or 0.01. Multiple comparisons of the means were conducted using the Tukey test. All data are expressed as mean ± SD.

## 4. Conclusions

Herein, we provide a strategy for heterologous overexpression of soluble and functional grouper interferon by taking advantage of bacterial strains and signal peptides. Recovery of recombinant proteins from periplasmic and extracellular spaces are often beneficial for protein purification, folding, and reducing associated costs. Soluble interferons were collected, purified, and evaluated in terms of immune responses and antiviral activity. This technology has great potential for commercial grouper interferon production as a promising product in aquaculture for treating viral infections.

## Figures and Tables

**Table 1:** Overexpression of recombinant fish IFNs in heterologous hosts.

IFNs	Source	Signal Peptides	Expression Host	Recombinant IFN Location	Yield ^a^ (mg/L Culture)	References
IFN	*D. rerio*	none	*E. coli*	Cytosolic	Insoluble	[18]
IFN-α	*E. septemfasciatus*	none	*E. coli*	Cytosolic	Soluble	[15]
IFN-α	*E. septemfasciatus*	none	*E. coli*	Cytosolic	Insoluble	[3]
IFN-α	*E. septemfasciatus*	SacB	*B. subtilis*	Extracellular	1.3	This study
IFN-α	*E. septemfasciatus*	PelB	*E. coli*	Periplasmic	6.0	This study

^a^ Yield of soluble recombinant IFNs.
